# The moderating effect of social support on the effectiveness of a web-based, computer-tailored physical activity intervention for older adults

**DOI:** 10.1177/13591053241241840

**Published:** 2024-04-15

**Authors:** Stephanie J Alley, Stephanie Schoeppe, Hayley Moore, Quyen G To, Jannique van Uffelen, Felix Parker, Mitch J Duncan, Anthony Schneiders, Corneel Vandelanotte

**Affiliations:** 1 Appleton Institute, School of Health, Medical and Applied Sciences, Central Queensland University, Australia; 2 RMIT, Vietnam; 3 Department of Movement Sciences, KU Leuven, Belgium; 4 School of Medicine & Public Health, College of Health, Medicine, and Wellbeing, The University of Newcastle, Australia; 5 School of Health, Medical and Applied Sciences, Central Queensland University, Australia

**Keywords:** behavioural medicine, e-health, health behaviour, older person, physical activity, social support, tailored advice, web-based

## Abstract

This study aimed to assess the moderating effect of social support on the effectiveness of a web-based, computer-tailored physical activity intervention for older adults. In the *Active for Life* trial, 243 inactive adults aged 65+ years were randomised into: (1) tailoring + Fitbit (*n* = 78), (2) tailoring-only (*n* = 96) or (3) control (*n* = 69). For the current study, participants were categorised as having higher (*n* = 146) or lower (*n* = 97) social support based on the Duke Social Support Index (DSSI_10). Moderate-to-vigorous physical activity (MVPA) was measured through accelerometers at baseline and post-intervention. A linear mixed model analysis demonstrated that among participants with lower social support, the tailoring + Fitbit participants, but not the tailoring only participants increased their MVPA more than the control. Among participants with higher social support, no differences in MVPA changes were observed between groups. Web-based computer-tailored interventions with Fitbit integration may be more effective in older adults with lower levels of social support.

## Introduction

Physical activity improves health and wellbeing throughout the ageing process (Bauman et al., 2016; Hupin et al., 2015). Specifically, physical activity is associated with lowering the risk of chronic health issues such as hypertension, type II diabetes and coronary heart disease (Bauman et al., 2016). Physical activity also improves mobility, sleep and mental health and reduces the risk of falls and age-related cognitive decline (Bauman et al., 2016). For those aged 65 years and over, the Australian Government Department of Health’s 2021 national physical activity guidelines recommend 30 minutes of moderate intensity physical activity on most days (Australian Government Department of Health, 2021). However, almost three quarters (74%) of older Australians, are not meeting these physical activity guidelines (Australian Bureau of Statistics, 2022). Therefore, it is vital to investigate population-based approaches to increase physical activity in older adults, so they can maintain a good quality of life with less reliance on the healthcare system (Australian Institute of Health and Welfare, 2020; Bauman et al., 2016).

The majority of older Australians are now using the internet daily (Australian Bureau of Statistics, 2020) and web-based physical activity interventions show promise as an effective intervention strategy in this age group (Jonkman et al., 2018; Muellmann et al., 2018; Stockwell et al., 2019). Web-based interventions can deliver personally tailored physical activity advice at low cost and without geographical constraints (Muellmann et al., 2019). Tailored advice has potential for older adults who have diverse characteristics that physical activity advice can be tailored to (e.g. current physical activity, interests, environment, health) (Volders et al., 2020). Limited studies have investigated the effectiveness of computer-tailored interventions in older adults, and those that have demonstrate mixed results (Muellmann et al., 2019; Van Dyck et al., 2016).

Recent computer-tailored interventions have also used objectively measured physical activity data from activity trackers to inform the computer-tailored physical activity advice in combination with survey data (e.g. health, barriers, benefits, environment) (Muellmann et al., 2019; Vandelanotte et al., 2018). Limited research suggests computer-tailored advice based on activity tracker data improves perceived credibility and effectiveness of the advice in adults 18+ years of age and is well received in older adults (Muellmann et al., 2019; Vandelanotte et al., 2018).

Behaviour change theories including social cognitive theory (Bandura, 1986), self-determination theory (Deci and Ryan, 1985) and the theory of planned behaviour (Ajzen, 1998) propose that a person’s social environment is an important construct for behaviour change and maintenance (Ajzen, 1998; Bandura, 1986; Deci and Ryan, 1985). A person’s social environment includes both social support and social norms (i.e. our group of friends are physically active) of a person’s community, which both influence physical activity behaviour (Rhodes et al., 2019; Samdal et al., 2017). Social support has been described as ‘support accessible to an individual through social ties to other individuals, groups, and the larger community’ (Lin et al., 1979: 109) whilst social norms refer to ‘the beliefs other people have about what other people expect or encourage others to do’ (Ball et al., 2010: 2). Social support specific to physical activity (e.g. inviting a friend to a community exercise class) is understood to have the strongest influence on physical activity (Lindsay Smith et al., 2017), however research also demonstrates that the more socially connected older adults are in general, the more physically active they are (Loprinzi and Joyner, 2016).

Despite the potential of computer-tailored physical activity interventions in older adults, such online interventions do not offer traditional social support with the presence of ‘real’ people (Alley et al., 2022). However, social support is an important component in helping older adults increase their physical activity (Lindsay Smith et al., 2017; Loprinzi and Joyner, 2016). It is possible that a web-based intervention providing tailored physical activity advice would be more effective in older adults with higher existing social support from their friends and family networks they can draw on (Loprinzi and Joyner, 2016; Resnick et al., 2002). Conversely, such interventions which do not directly provide social support may be less effective in those without existing social support to help them increase their activity (Yardley et al., 2016). Further, participants with higher levels of social support may be more engaged and satisfied with the intervention content, as they have a strong social network they can use to support them in implementing the tailored advice and action plans to change their physical activity behaviour (Loprinzi and Joyner, 2016; Resnick et al., 2002). Understanding how physical activity determinants such as social support influence the effectiveness of web-based physical activity interventions is necessary to inform future policies and targeted interventions strategies that promote healthy ageing.

This study was conducted as a secondary analysis of the *Active for Life* study, which was a randomised controlled trial examining the effectiveness of a computer-tailored web-based physical activity intervention for older adults with and without activity tracker (Fitbit) integration (Alley et al., 2022). Results demonstrated that the Fitbit and tailoring participants increased their Moderate to Vigorous Physical Activity (MVPA) from baseline to post-intervention by only 2 minutes per day, however this was statistically significant compared to the control group who decreased their MVPA by 7 minutes per day. The tailored advice only group decreased their MVPA by 1 minute per day which was not statistically significant compared to the control group (Alley et al., 2022). The main aim of this study was to assess the moderating effect of baseline general social support on changes in MVPA in older Australians receiving physical activity advice in a web-based computer-tailored physical activity intervention (Alley et al., 2019, 2022). The secondary aim of this study was to assess the moderating effect of baseline social support on older adults’ engagement and acceptability of a web-based computer-tailored physical activity intervention.

## Methods

### Study design

This study used data from a three-group randomised controlled trial that tested the effectiveness of the *Active for Life* computer-tailored intervention aiming to increase physical activity in older adults (Alley et al., 2019, 2022). The trial randomly assigned participants to one of three groups: (1) control, (2) tailored advice only, (3) tailored advice and Fitbit. Objective MVPA was measured at baseline and post-intervention (week 12) and participants attended a face-to-face appointment at baseline and post-intervention (week 12) to connect participants to the intervention and ensure data collection was complete. Participants also completed surveys at baseline and post-intervention (week 12) to assess demographics, health status, social support and acceptability of the intervention. Engagement with the intervention was tracked through the website and google analytics (e.g. time on site and sessions completed). Further information can be found in the *Active for Life* protocol paper and main outcomes paper (Alley et al., 2019, 2022).

### Participants

For the original *Active for Life* study, recruitment was conducted between April 2018 and March 2019 in Rockhampton (QLD), Bundaberg (QLD) and Adelaide (SA) Australia using multiple methods (e.g. emails lists, Facebook posts and advertisements, flyers and local newsletters).

Eligibility to participate in the study required adults to be aged 65 years or older, English speaking and able to access and confidently navigate the internet. They needed to demonstrate that they had no health issues (incl. heart conditions, joint problems and high blood pressure) that might affect their ability to safely increase their physical activity through the physical activity readiness questionnaire (PARQ) (Cardinal et al., 1996). If the PARQ identified a health issue, they could still participate in the study with approval from a GP. Those who were unable to attend the face-to-face appointments, had used an activity tracker during the previous 6 months, who were already participating in another physical activity programme (e.g. 10,000 steps) or were already meeting Australia’s physical activity guidelines (Australian Government Department of Health, 2021) were ineligible for the study.

### Procedures

*Active for Life* recruitment advertising guided respondents to a website, where they found further details of the study, including the information sheet and a screening survey to complete. Eligible participants were asked to complete a consent form and online survey questionnaire on the *Active for Life* website at baseline. Accelerometers measuring physical activity and sedentary behaviour at baseline and at post intervention (Week 12) were posted to participants to wear on their non-dominant wrist for seven consecutive days including sleeping and showering. Stratified randomisation was adopted to ensure an even spread across the study groups of men and women as well as participants aged under 75 years and over 75 years. During the initial appointment, participants were randomised using computer-automated block randomisation with block sizes of 15 and a 1:1:1 ratio. Intervention participants were then guided through the *Active for Life* website, and tailoring + Fitbit participants were shown how to use the accelerometer and Fitbit activity tracker and how to sync their data with the *Active for Life* website. Participants in the control group were given access to the intervention at the 24-week mark of the study. For the original study, ethics approval (approval number H16/12-321) was received from Central Queensland University Human Ethics Committee prior to commencement of data collection.

### The Active for Life intervention

The *Active for Life* intervention was a web-based programme with six modules of computer-tailored physical activity advice delivered fortnightly over 12 weeks. Based on participants’ responses to survey questions, the physical activity advice was personalised using if-then algorithms to select advice from a database of precomposed messages. The website-delivered physical activity advice was based on the theory of planned behaviour (Ajzen, 1998) and social cognitive theory (Bandura, 1986). Topics included physical activity recommendations, benefits and barriers, exercising safely, exercising with a chronic disease, sedentary behaviour, goal setting, action plans, self-efficacy, physical and social environments, rewards, forming habits and preventing relapse. The computer-tailored physical activity advice received by both the tailoring + Fitbit participants and the tailoring only participants encouraged participants to be active with a friend or family member, but the intervention did not specifically facilitate meet ups or any other form of social support. Participants could access strength and flexibility exercise plans, devised by a physiotherapist, aimed at beginner and intermediate abilities on the intervention website. Participants were also encouraged to use an action-planning tool on the website to decide upon the ‘what, where, when and with whom’ of their physical activity for the following fortnight. More detail of the intervention can be found in the study protocol paper (Alley et al., 2019).

#### Measures

Sociodemographic information was collected in an online survey. This included: age, sex (male, female), location (Bundaberg, Rockhampton, Adelaide), English as main language, marital status (single, in a relationship), education (secondary, technical college, university), employment status (full time, part time, not employed), chronic disease diagnoses (yes, no) and household income.

Social support was assessed in the online survey at baseline using the Abbreviated Duke Social Support Index (DSSI_10), a 10-item instrument designed to ascertain a person’s level of social support (Pachana et al., 2008). The DSSI_10 asked participants to report how many people they depend on and the frequency of face-to-face, telephone and meeting-type social interactions. There were four questions related to social interaction (e.g. about how often did you go to meetings of clubs, religious meetings or other groups that you belong to in the past week?) and seven questions to rate relationship satisfaction (e.g. when you are talking with your family and friends, do you feel you are being listened to?). Evidence for internal consistency reliability (Cronbach’s Alpha = 0.60 for the social interaction subscale and 0.80 for the social satisfaction subscale) has been found using the DSSI_10 in older adults (Pachana et al., 2008). Possible scores range from 10 to 30 with higher scores representing higher social support. There is no established cut point of the DSSI_10 for high social support, however in line with previous studies (Gerlach et al., 2017) we used ≤23 as the cut point for low social support based on population data collected through the National Institute of Mental Health Epidemiologic Catchment Area (ECA) survey (Hughes et al., 1993).

Physical activity and sedentary behaviour were measured objectively at baseline and post intervention (Week 12) using the ActiGraph GT9X wrist-worn accelerometer. Vector magnitude cut points found by Kamada et al. (2016) were used to distinguish between sedentary behaviour (<2000), light physical activity (2000–8249) and MVPA (≥8250). The minimum wear time cut point for inclusion of accelerometer data in the analyses was set at 16 hours on at least 5 days each week, as determined by Kamada et al. (2016).

Intervention engagement was assessed throughout the 12-week intervention via website data (module completion) and Google Analytics measures (time spent on the website, and time spent on the action planning feature).

Acceptability of the tailored advice was measured at week 12 through nine items based on past research (Vandelanotte et al., 2018) but adapted for this study. Questions included ‘the physical activity advice was (a) interesting, (b) credible, (c) easy to understand, (d) personally relevant, (e) held me accountable’, (f) ‘through the advice I learned something new about my own physical activity’, (g) ‘too much physical activity was provided’, (h) ‘I have used the advice to become more active’ and (i) ‘I have changed my opinion about physical activity because of this program’. The items were on a 7-point Likert scale ranging from ‘strongly agree’ to ‘strongly disagree’. The individual questions were collapsed into two categories for reporting and analysis: (1) agree and (2) disagree or neutral. The agree category included ‘strongly agree’, ‘agree’ and ‘somewhat agree’ responses. The disagree or neutral category included ‘neither agree nor disagree’, ‘somewhat disagree’, ‘disagree’ and ‘strongly disagree’ responses. An average acceptability rating was calculated for each participant by averaging ratings of the nine acceptability statements (ratings of negative statements were reverse scored).

### Data analysis

Means (95% CI) were calculated for objectively measured MVPA at baseline and post-intervention (week 12) and intervention engagement and acceptability measures. These were split by participants with lower and higher social support. A generalised linear mixed model with a gamma distribution and loglink was used for MVPA as it was positively skewed. The model included a fixed effect for study group (tailoring + Fitbit, tailoring only, control), social support (higher, lower), time (baseline, post-intervention), a three-way interaction term (study group × social support × time) and a random intercept to account for repeated measures on individuals. The model was adjusted for baseline MVPA. Means ratios were presented for each level of moderators. Two-way Analysis of Variances (ANOVA) were conducted on the engagement measures (time on site, module completion and time on action plans) and intervention acceptability ratings. The model included main effects of social support (lower social support, higher social support) and intervention group (tailoring + Fitbit, tailoring only) and a two-way interaction between social support and intervention group. All *p*-values were two sided and significance was set at <0.05. SPSS v26 was used for all analyses.

## Results

Table 1 shows the sociodemographic characteristics of the *Active for Life* participants at baseline. Of the 243 participants, 79% were female, 62% were from Adelaide, 95% spoke English as their first language, 71% were in a relationship and 52% had a university education. About 73% of participants were not employed (including retired) and 44% of participants had a low household income (i.e. <AUD40,000). Over a third had a chronic disease diagnosis (37%) and the average age was 69 years (SD 4.32; range 65–98 years). Table 1 demonstrates that the sociodemographic characteristics were similar between participants with higher and lower social support and the main outcomes paper (Alley et al., 2022) reports that the sociodemographic characteristics were similar between study groups and between study completers and study dropouts. Study attrition was 31% (46/146) in high social support participants and 32% (31/97) in low social support particpants.

**Table 1.** Sociodemographic characteristics of participants at baseline.

	All participants (*N* = 243)	Lower social support (*n* = 138)	Higher social support (*n* = 105)	Comparison χ^2^ (*df*), *p*	Comparison *t* (*df*), *p*
Sex, *n* (%)					
Male	52 (21.4)	30 (21.7)	22 (21.0)		
Female	191 (78.6)	108 (78.3)	83 (79.0)	0.022 (1), *p* = 0.882	
Location, *n* (%)					
Rockhampton	74 (30.5)	44 (31.9)	30 (28.6)		
Bundaberg	18 (7.4)	11 (8.0)	7 (6.7)		
Adelaide	151 (62.1)	83 (60.1)	68 (64.8)	0.556 (2), *p* = 0.757	
Primary language, *n* (%)					
English	231 (95.1)	133 (96.4)	98 (93.3)		
Other	12 (4.9)	5 (3.6)	7 (6.7)	1.177 (1), *p* = 0.278	
Marital status, *n* (%)					
Single	71 (29.2)	40 (29.0)	31 (29.5)		
Married/de facto	172 (70.8)	98 (71.0)	74 (70.5)	0.008 (1), *p* = 0.927	
Education level, *n* (%)					
Secondary School	61 (25.1)	34 (24.6)	27 (25.7)		
Technical collage	56 (23.0)	32 (23.2)	24 (22.9)		
University	126 (51.9)	72 (52.2)	54 (51.4)	0.037 (2), *p* = 0.982	
Employment, *n* (%)					
Full time	22 (9.1)	16 (11.6)	6 (5.7)		
Part time or casual	43 (17.7)	24 (17.4)	19 (18.1)		
Not employed	178 (73.3)	98 (71.0)	80 (76.2)	2.512 (2), *p* = 0.285	
Chronic disease was present, *n* (%)					
Yes	89 (36.6)	51 (37.0)	38 (36.2)		
No	154 (63.4)	87 (63.0)	67 (63.8)	0.015 (1), *p* = 0.902	
Income, *n* (%)^a^					
104,000+	24 (12.7)	14 (13.0)	10 (12.3)		
65,000–103,999	29 (15.3)	19 (17.6)	10 (12.3)		
41,000–64,999	52 (27.5)	27 (25.0)	25 (30.9)		
<40,000	84 (44.4)	48 (44.4)	36 (44.4)	1.423 (3), *p* = 0.700	
Age in years, *M* (SD)	68.84 (3.85)	68.88 (3.61)	69.93 (5.07)		*t* = −1.884 (1, 241), *p* = 0.610
MVPA, *M* (SD)	17.08 (19.50)	16.85 (20.86)	17.38 (17.67)		*t* = (1, 207), −0.194, *p* = 0.847

*M*: mean; SD: standard deviation; MVPA: Moderate to Vigorous Physical Activity.

a Income missing (did not wish to disclose): *n* = 54.

Table 2 presents the descriptive data for MVPA by time, intervention group and social support group. In participants with lower social support, both tailoring only and Fitbit + tailoring participants increased their MVPA from baseline to post intervention (Week 12) whilst the control group decreased their physical activity. In comparison, all participants with higher social support, regardless of group, decreased their MVPA per day from baseline to post intervention.

**Table 2.** MVPA and sedentary minutes by study groups, social support and time.

	Control (*n* = 59)			Tailoring only (*n* = 81)			Fitbit + tailoring (*n* = 69)		
	*N*	Mean (95% CI)	Mean ratio (95% CI)	*N*	Mean (95% CI)	Mean ratio (95% CI)	*N*	Mean (95% CI)	Mean ratio (95% CI)
Social support									
Low (*n* = 119)									
Baseline	31	20.78 (12.06, 35.80)	1	48	17.94 (13.83, 23.27)	1	40	12.50 (9.47, 16.49)	1
Post intervention	26	13.18 (9.83, 17.67)	1	25	18.38 (13.21, 25.55)	1.61 (0.79, 3.28), *p* = 0.186	30	18.15 (11.86, 27.76)	2.29 (1.05, 4.99), *p* = 0.037*
High (*n* = 90)									
Baseline	28	15.43 (10.54, 22.59)	1	33	18.76 (14.36, 24.51)	1	29	14.28 (10.29, 19.82)	1
Post intervention	15	8.08 (4.40, 14.83)	1	24	14.65 (10.21, 21.03)	1.49 (0.73, 3.03), *p* = 0.267	21	12.06 (7.00, 20.77)	1.61 (0.71, 3.64), *p* = 0.249

Of the 243 participants, 34 participants were excluded due to insufficient accelerometer data at baseline. Model is adjusted for baseline MVPA. Model effects: three-way interaction = *p* = 0.270.

MVPA: moderate to vigorous physical activity; mins/day: minutes per day.

* *p*  <  0.05.

The overall moderating effect of social support on MVPA changes between groups (Fitbit + tailoring, Fitbit only, control), was not statistically significant (*p* = 0.270). However, the Fitbit + tailoring participants with lower social support increased their MVPA significantly more than the control participants with lower social support. Whilst the tailoring only participants with lower social support also increased their MVPA more than the control participants with lower social support, this was not significant. No increase in MVPA was observed for either Fitbit + tailoring or tailoring only participants with higher social support compared to control participants with higher social support.

Table 3 presents descriptive statistics of intervention engagement (time on site, module completion and time on action plans) and average intervention acceptability ratings. Table 3 also presents comparisons of intervention engagement and average intervention acceptability ratings by social support group (lower social support, higher social support) and intervention group (tailoring + Fitbit, tailoring only). No significant differences on either engagement or acceptability were observed between participants with high and low social support, nor between tailoring + Fitbit participants or tailoring only participants. Further, no interaction effects were observed (social support × intervention group) for engagement or acceptability.

**Table 3.** Intervention engagement and acceptability by intervention group and social support.

	Descriptives				Comparison					
	Lower social support		Higher social support		Main effects				Interaction	
	Tailoring + Fitbit	Tailoring only	Tailoring + Fitbit	Tailoring only	High vs low social support		Tailoring + Fitbit vs tailoring only		Social support × intervention group	
	*M* (SD)^a^	*M* (SD)	*M* (SD)	*M* (SD)	*F* ^b^	*p* ^c^	*F*	*p*	*F*	*p*
Time on site (minutes (0–12 weeks)) *n* = 173^a^	132.26 (68.59)	131.44 (68.78)	126.89 (54.84)	140.24 (74.12)	0.018	0.892	0.244	0.622	0.312	0.578
Module completion (min = 0, max = 6) *n* = 174	5.62 (0.91)	5.61 (1.09)	5.75 (0.61)	5.88 (0.59)	1.584	0.211	0.130	0.719	0.229	0.633
Time on action plans (minutes 0–12 weeks) *n* = 173^a^	20.09 (16.60)	19.11 (15.78)	19.94 (11.45)	18.71 (12.86)	0.010	0.921	0.161	0.698	0.002	0.962
Advice acceptability average rating (min = 1, max = 7) *n* = 115	5.75 (0.82)	5.75 (0.71)	5.72 (0.80)	5.52 (0.95)	0.895	0.346	0.541	0.464	0.252	0.616

a Mean (standard deviation).

b *F* statistic.

c *p* Value.

## Discussion

The aim of this study was to assess the moderating effect of baseline social support on changes in MVPA in older Australians receiving physical activity advice in a computer-tailored, web-based intervention (Alley et al., 2022). It was hypothesised that participants with higher social support at baseline would be in a better position to increase their physical activity over the course of the computer-tailored intervention. This was based on past research demonstrating greater increases in physical activity in older adults with more social support (Kouvonen et al., 2012; Samdal et al., 2017). The findings showed that there was no overall moderating effect of social support on physical activity changes between tailoring + Fitbit and tailoring only groups compared to control from baseline to post intervention (Week 12). However, in participants with lower social support, tailoring + Fitbit participants increased their MVPA by +5 minutes per day which was significantly more than the control group who decreased their MVPA by −5 minutes per day. The tailoring only group only increased their MVPA by 1 minute per day which was not significantly different than the control group. In comparison, participants with higher social support decreased their MVPA across all study groups and no significant differences were observed between the tailoring + Fitbit group (−3 minutes) or the tailoring only group (−4 minutes) compared to the control group (−10 minutes).

Although the hypothesis was not supported, the finding that the participants with lower social support but not higher social support who received the tailored advice + Fitbit increased their physical activity compared to the control group is important. This is not in line with past research demonstrating that people, particularly older adults with higher levels of social support have greater improvements in physical activity (Kouvonen et al., 2012; Samdal et al., 2017). However, no studies have investigated how physical activity changes following a web-based intervention differs between older adults with higher and lower social support. Ratz et al. (2021) found that older adults who were socially inactive were much more likely to drop out of a web-based computer-tailored physical activity intervention. However, the intervention delivered in Ratz et al. (2021) also included weekly group exercises. It is possible that participants with lower social support are less suited to face-to-face or group physical activity interventions.

In contrast, people with lower social support may have been well suited to accessing the web-based intervention when and where they preferred and planning their own physical activity throughout the intervention. Web-based interventions could also ameliorate other barriers to physical activity for older adults with lower social support, such as having too few friends to exercise with, lack of transport or lack of awareness of group or expert lead physical activity classes (Moschny et al., 2011). The ability of web-based physical activity interventions to overcome these barriers could explain the increase in physical activity that was observed in intervention participants with lower social support. The fact that participants with lower social support in the tailoring + Fitbit group, but not the tailoring only group, increased their physical activity compared to the control, suggests that it could have been the Fitbit component of the intervention that drove intervention effectiveness in this group. It is possible that the participants with low social support may have particularly benefited from self-monitoring their physical activity through an activity tracker, or receiving tailored advice based on their activity tracker data. Overall, these findings suggest that web-based programmes that deliver computer-tailored physical activity advice with fit-bit integration show promise for older adults with lower social support.

The secondary aim of this study was to assess the moderating effect of baseline social support on older adults’ engagement and acceptability of a web-based computer-tailored physical activity intervention (Alley et al., 2022). It was hypothesised that participants with higher social support will have improved engagement and acceptability of the computer-tailored physical activity intervention as they have a support network they can draw on when applying the tailored behaviour change advice (Corbett et al., 2018; Loprinzi and Joyner, 2016). However, when considering the main finding that participants with lower and not higher social support in the tailoring + Fitbit group increased their physical activity compared to the control, it would follow that participants with lower social support may be more suited to the computer-tailored intervention and therefore be more engaged and rate it higher compared to participants with higher levels of social support. This was however not supported by the results which demonstrated no substantial differences between participants with lower and higher social support on intervention engagement or acceptability.

### Strengths and limitations

The strengths of this study include the objective measurement of physical activity at baseline and post intervention in the *Active for Life* study. The *Active for Life* study also included randomisation to intervention and control groups and had similar attrition rate to other studies (Kelders et al., 2011) examining web-based interventions.

One limitation of this study was that the DSSI_10 used in the *Active for Life* study to measure social support solely assessed all social interactions and satisfaction with social interactions broadly, but not other social support components specific to physical activity, such as emotional support, appraisal support and instrumental support (e.g. family member driving someone to an exercise class) (Resnick et al., 2002). As such, future research could use an instrument that measures social support specific to physical activity. Further, participants were categorised into lower and higher levels of social support based on population data collected through the National Institute of Mental Health Epidemiologic Catchment Area (ECA) survey (Hughes et al., 1993) which has been used as a cut point to identify low social support in other studies (Gerlach et al., 2017). However, most participants clustered around this cut point and few participants actually had very low social support indicating loneliness or isolation. Future studies could recruit participants with low or very low levels of social support to determine the effectiveness of computer-tailored physical activity interventions in this group. Another limitation was that the study sample was powered for main effects (i.e. physical activity and sedentary behaviour changes between intervention groups from baseline to post-intervention), not for moderation analyses which splits the sample further. More research with larger sample sizes is required.

## Conclusion

This study assessed the moderating effect of social support on MVPA and sedentary behaviour in older Australians receiving the computer-tailored, web-based intervention. The only group who significantly increased their physical activity were lower social support participants in the tailored advice + Fitbit group. Therefore, web-based physical activity programmes with computer-tailored advice and activity tracker integration may be suited to participants with lower levels of general social support. Further research in larger samples of participants with lower levels of social support, which measures additional social support constructs (e.g. emotional and instrumental social support specific to physical activity) is needed.

## Supplemental Material

sj-docx-1-hpq-10.1177_13591053241241840 – Supplemental material for The moderating effect of social support on the effectiveness of a web-based, computer-tailored physical activity intervention for older adults Supplemental material, sj-docx-1-hpq-10.1177_13591053241241840 for The moderating effect of social support on the effectiveness of a web-based, computer-tailored physical activity intervention for older adults by Stephanie J Alley, Stephanie Schoeppe, Hayley Moore, Quyen G To, Jannique van Uffelen, Felix Parker, Mitch J Duncan, Anthony Schneiders and Corneel Vandelanotte in Journal of Health Psychology

sj-sps-2-hpq-10.1177_13591053241241840 – Supplemental material for The moderating effect of social support on the effectiveness of a web-based, computer-tailored physical activity intervention for older adults Supplemental material, sj-sps-2-hpq-10.1177_13591053241241840 for The moderating effect of social support on the effectiveness of a web-based, computer-tailored physical activity intervention for older adults by Stephanie J Alley, Stephanie Schoeppe, Hayley Moore, Quyen G To, Jannique van Uffelen, Felix Parker, Mitch J Duncan, Anthony Schneiders and Corneel Vandelanotte in Journal of Health Psychology

sj-spv-3-hpq-10.1177_13591053241241840 – Supplemental material for The moderating effect of social support on the effectiveness of a web-based, computer-tailored physical activity intervention for older adults Supplemental material, sj-spv-3-hpq-10.1177_13591053241241840 for The moderating effect of social support on the effectiveness of a web-based, computer-tailored physical activity intervention for older adults by Stephanie J Alley, Stephanie Schoeppe, Hayley Moore, Quyen G To, Jannique van Uffelen, Felix Parker, Mitch J Duncan, Anthony Schneiders and Corneel Vandelanotte in Journal of Health Psychology

sj-sav-4-hpq-10.1177_13591053241241840 – Supplemental material for The moderating effect of social support on the effectiveness of a web-based, computer-tailored physical activity intervention for older adults Supplemental material, sj-sav-4-hpq-10.1177_13591053241241840 for The moderating effect of social support on the effectiveness of a web-based, computer-tailored physical activity intervention for older adults by Stephanie J Alley, Stephanie Schoeppe, Hayley Moore, Quyen G To, Jannique van Uffelen, Felix Parker, Mitch J Duncan, Anthony Schneiders and Corneel Vandelanotte in Journal of Health Psychology
