# Supplementary material for: The moderating effect of social support on the effectiveness of a web-based, computer-tailored physical activity intervention for older adults
Source: J Health Psychol. 2024 Apr 15;30(6):1306–18. doi: 10.1177/13591053241241840 (PMC12052858; doi:10.1177/13591053241241840)
Supplement: sj-docx-1-hpq-10.1177_13591053241241840 – Supplemental material for The moderating effect of social support on the effectiveness of a web-based, computer-tailored physical activity intervention for older adults [file sj-docx-1-hpq-10.1177_13591053241241840.docx]

**The moderating effect of social support on the effectiveness of a web-based, computer-tailored physical activity intervention for older adults.**

**Data files (de-identified)**

Please find included 3 data files to replicate analysis:

- Main data file in SPSS (Data_File_Social Support_revision.sav)
  - Variable descriptions can be found under ‘label’
- Data syntax in SPSS (Data Syntax_ Social Support_revision.sps)
  - Syntax includes main outcomes analysis
- Output file in SPSS (Data Output_ Social Support_revision.spv)
  - All output produced by SPSS.
